# Ten Simple Rules for Selecting a Bio-ontology

**DOI:** 10.1371/journal.pcbi.1004743

**Published:** 2016-02-11

**Authors:** James Malone, Robert Stevens, Simon Jupp, Tom Hancocks, Helen Parkinson, Cath Brooksbank

**Affiliations:** 1 European Bioinformatics Institute (EMBL-EBI), Cambridge, United Kingdom; 2 School of Computer Science, University of Manchester, Manchester, United Kingdom; UNITED STATES

## Introduction

Biologists and bioinformaticians now look to ontologies or software that uses ontologies as a means of standardising the way data are described, queried, and interpreted. Ontologies can be used for the annotation and curation of experimental datasets and, in data sharing, both within and beyond the confines of individual labs, organizations, and communities. Bio-ontologies are also commonly used in methods of analysis, particularly in gene set enrichment analysis [1], using ontologies such as the Gene Ontology. With modern high-throughput data-generation technologies, there is now, more than ever, a need to integrate data from these and other sources, and there is a concomitant need for ontologies—raising the question of how to choose a bio-ontology.

Over the past decade, a community has grown up around the success of efforts to harmonise the semantic description of biological entities, with ontologies exemplified in the emergence of the Open Biological and Biomedical Ontologies (OBO) Foundry [2]. These efforts were first led by the aforementioned Gene Ontology [3] and have expanded to ontologies that describe a significant range of the primary areas of biology and its science. Exploring bio-ontologies through browsers such as the Ontology Lookup Service [4] at the European Bioinformatics Institute and BioPortal [5] at the National Center for Biomedical Ontology (NCBO)—whose existence is itself a measure of the community size—shows there are over 400 ontologies containing, collectively, over 5 million classes (by classes, we mean ontological terms together with their associated descriptions and synonyms). These ontologies cover areas such as diseases [6], phenotypes [7], anatomy [8], experimental conditions [9,10], cell types [11], and bioinformatics software [12].

There are now many ontologies from which to choose, but which ontology should be chosen? In order to answer this question, we present ten simple rules that should help to guide the choice of a bio-ontology. The rules are designed to be useful for those wishing consume a bio-ontology. Users of bio-ontologies are varied in their profile and include data curators, application developers, and, of course, ontology developers who may be consuming part of an ontology in their own work.

## Rule 1: The Ontology Should Be about a Specific Domain of Knowledge

Specifically, an ontology should provide coverage for the area it claims to describe. Although almost no ontology is complete, you should aim to find an ontology that describes a considerable amount of the area to which it lays claim. It should also describe the field of interest in such a way that extensions to cover missing areas are possible without a major rewriting of the ontology. Missing terms are to be expected, but if the ontology is missing large areas that are key to describing the domain, then it may not be a suitable ontology. For instance, a disease ontology that does not include cancers would be inadequate if the diseases that you were aiming to describe included cancers. Furthermore, even if you personally don’t have any cancer data to describe, you need to consider the notion that a disease ontology with such a large gap in it is unlikely to gain wide community adoption. Conversely, if an ontology claims to only describe a specific subset of a domain, or even just a local application, then it should do so appropriately and should not be considered an unsuitable ontology simply because it has a more limited scope; it may be less useful to the wider world, but this does not make it a bad ontology. One computational service that can help a user estimate whether or not an ontology can provide coverage is the NCBO Ontology Recommender [13]. Recommender measures whether an ontology from the NCBO BioPortal matches a given set of text based on a measure of coverage, which can help to inform whether a given ontology contains the terms a user might be expecting.

## Rule 2: The Ontology Should Reflect Current Understanding of Biological Systems

Unless the aim of the ontology is to capture a historic viewpoint (a legitimate objective), then it should reflect current science, or at least not contradict it. For instance, the old dogma of DNA to RNA without feedback would no longer be accepted in modern biology. It is better to make statements that are too broad but remain correct rather than make specific statements that are wrong. The correctness of an ontology is often evaluated using techniques such as competency questions. [14]. Competency questions are queries that are required to be answerable by an ontology, with the returned answers thus demonstrating whether or not an ontology is giving correct, expected answers. In the simplest form, a user may ask for subtypes of a class, e.g., “What are the subclasses of fat cell?”, but this can also be more complex depending upon needs, e.g., “Which cell lines are derived from human, epithelial cells and are taken from melanoma samples?” Correct answers suggest the ontology is reflecting current science correctly.

## Rule 3: The Ontology Classes and Relationships Should Persist

One of the primary use cases of an ontology is to describe biomedical data through annotations; disconnecting the descriptions of this data from their semantics through the deletion of the ontology identifiers undermines the advantage of using ontologies in the first instance. This is crucial if these ontologies’ annotations are being used for data sharing, integration, or analysis. Identifiers in most biomedical ontologies are formed using accessioned IDs rather than textual labels, with the intent of removing potential ID clashes and decoupling the textual part of a class (i.e., the label) from the identifier referring to it. This has the advantage of enabling small modifications to a class label without affecting the class identifier, where the class is still referring to the same entity. In cases in which the identifier is a Uniform Resource Identifier (URI), these should resolve to provide both human-readable and machine-interpretable information. Services like Identifiers.org provide a URI resolution service for many biomedical ontologies. Identifiers should be maintained, and if it is necessary to remove a class, it should be labelled as “obsolete” rather than simply deleted. Maintaining this audit trail is the sign of a well-managed ontology—deleting identifiers is the sign of a poorly managed ontology.

## Rule 4: Classes Should Contain Textual Definitions

This is crucial for users who come to an ontology trying to understand what a particular class is describing. It may, on occasion, be obvious—“*Homo sapiens*,” for instance. On others, it is critical—a cell line named “Bas666” could be difficult to interpret. An additional sign of a suitable ontology is that it contains appropriate synonyms (e.g., “human” for “*Homo sapiens*”) and related alternative terms (e.g., in Gene Ontology, use of narrow synonyms such as “type I programmed cell death” for the label “apoptotic process”), since language can differ between communities and specialities even though the underlying class being described is the same thing. As well as textual definitions, many ontologies also contain logical descriptions of the class that are amenable to computational interpretation. These descriptions use rules, or “axioms,” to relate a class to other classes, such as describing that a heart is part of the cardiovascular system. Whether or not such computational aspects are necessary for a particular use case should form part of the decision when selecting a bio-ontology.

## Rule 5: Textual Definitions Should Be Written for Domain Experts

Creators of ontologies often fall into the trap of defining classes using ontology jargon (often philosophical in nature). This may make them understandable to ontologists and/or philosophers, but this is not useful if the language used means nothing to the ontology’s user community. A good ontology will reflect commonly used nomenclature in naming classes within it. Similarly, textual definitions should also reflect common language used in the biological domain. Textual definitions and labels that include ontology jargon are the sign of an unsuitable ontology. Ontologists should accurately describe the biomedical domain without modifying it.

## Rule 6: The Ontology Should Be Developed by the Community but Not Incapacitated by It

Reflecting current science is a difficult task, given the growing knowledge of the breadth and depth of entities in the domain. Gaining community consensus is a noble cause and should help to reflect current science correctly and enhance opportunities for wide adoption of an ontology. It is also almost always better to work towards getting entities added into an existing ontology that is supported by a community rather than inventing a new ontology. Engaging with the community, however, should not deflect from the task of developing an ontology. Decision making—should a user ask for a new class, for example—should not take months while consensus is obtained. Similarly, a lone gatekeeper making all the decisions about what happens within an ontology is also a bad sign. Most ontologies will have a public forum for dealing with user requests, and looking at mailing list archives or issue trackers (e.g., Gene Ontology http://geneontology.org/page/go-mailing-lists) will provide insight on how the ontology is being developed.

Another aspect of collaborating is that of compromise. Typically, everyone has an opinion on the science in which they are interested, and typically they don’t all align, so there is an element of compromise to selecting an ontology. A favoured label might not exist in the ontology, but rejecting wholesale a community-developed ontology in favour of inventing a de novo artefact with one’s own favourite labels is not always the best option. As above though, there are circumstances under which this may be ultimately the better option; here, the balance is in weighing up requirements and making a judgement based on what is most important. Wider integration with a community is a good thing when it works.

## Rule 7: The Ontology Should Be under Active Development

An ontology should have a dedicated presence on the web, such as a project website that provides information on how to contact the developers and contribute to the ontology. Any associated mailing lists or version control systems can be used to gauge recent activity on the ontology. Recent work [15] has shown that it is possible to describe how actively an ontology is maintained and in what way it is being modified. In general, an ontology that is not actively developed and has not been updated for many months or years is unlikely to respond to new requirements should they occur.

## Rule 8: Previous Versions Should Be Available

Given the changing nature of data and, hopefully, of the ontology as it updates to reflect current knowledge, data annotations can become out of date. An ontology should provide a clear versioning and release policy. It is important to be able to access older versions of an ontology so the context of data descriptions can be understood. This also relates to Rule 3 about not deleting ontology classes, and in turn both rules relate to enabling reproducibility of data analysis. Being unable to trace provenance of data annotations made with an older version of an ontology is a barrier to future reproducibility; selecting an ontology that maintains previous versions is therefore an important consideration.

## Rule 9: Open Data Requires Open Ontologies

An important consideration is whether or not the ontology is being selected for use with open data with the intention of wider sharing. Using an ontology that is restrictive in licensing can also have an impact on the data described with such an ontology, restricting access to the semantics which are necessary to understand it. If data sharing is the aim, then using ontologies with permissive licenses should be a priority. Permissive licenses, such as those developed by Creative Commons (https://creativecommons.org/licenses/), can be used to communicate both how the work (ontology) can be exploited and how attribution should be given. One of the important outcomes of the standardization efforts from the OBO Foundry has been the widespread use of the OBO Relation Ontology (RO), an open ontology of biomedical relationships. The widespread use of RO has led to a de facto standard in much of the bio-ontology world, which has had positive implications on integration of resources, facilitated by the open license with which the RO is released.

## Rule 10: Sometimes an Ontology Is Not Needed at All

Ontologies provide a means of “knowing” what is being described in a data set. There is, however, more than one way to capture such knowledge. Before embarking on using or indeed making a bio-ontology, you need to decide whether an ontology is really what is needed. In the broadest terms, we are talking about knowledge organisation systems of which there are numerous types of useful resources: glossaries, taxonomies, thesauri, ontologies, and terminologies. As a growing discipline, there is a temptation to suggest that using biomedical ontologies will offer some advantage. Ontologies offer advantages over other knowledge systems—they enable both computational use and human understanding, they can contain multiple classification axes of classes as well as formal descriptions of how classes relate to one another, and can include rich vocabularies of labels, synonyms, and textual definitions. If these are desirable selection criteria, then an ontology should be considered. Ontologies do also come with computational overheads, however, and can be complex to understand. Languages such as the Web Ontology Language (OWL) [16] utilise description logics, which are technically challenging. Other resources such as a vocabulary do not offer the sorts of classification and rich computational descriptions of an ontology but are often much simpler to understand. Let your requirements guide you; ontologies are not a panacea—sometimes one isn’t needed at all.

## Conclusion

Bio-ontologies represent an important tool for describing metadata, an increasingly important consideration as the scientific community aims for open, reusable data. It is perhaps no surprise then that in the Ten Simple Rules for the Care and Feeding of Scientific Data [17], the word “metadata” appeared 11 times. The choice of which ontology to pick and even when to use one is not always straightforward, as demonstrated by the number of times the authors are asked to recommend a particular ontology for a given problem. The single most important consideration in selecting a bio-ontology is to understand requirements first before deciding to engage with a particular ontology or indeed before minting one’s own ontology. By identifying needs and selecting current ontologies using the above rules, it is possible to reach a conclusion as to whether or not a resource is useful to a given user. Moreover, reusing ontologies that similarly satisfy another user’s needs helps to spread the burden of development across the community and ensure we don’t end up with islands of metadata, undermining the efforts of openness and sharing.
